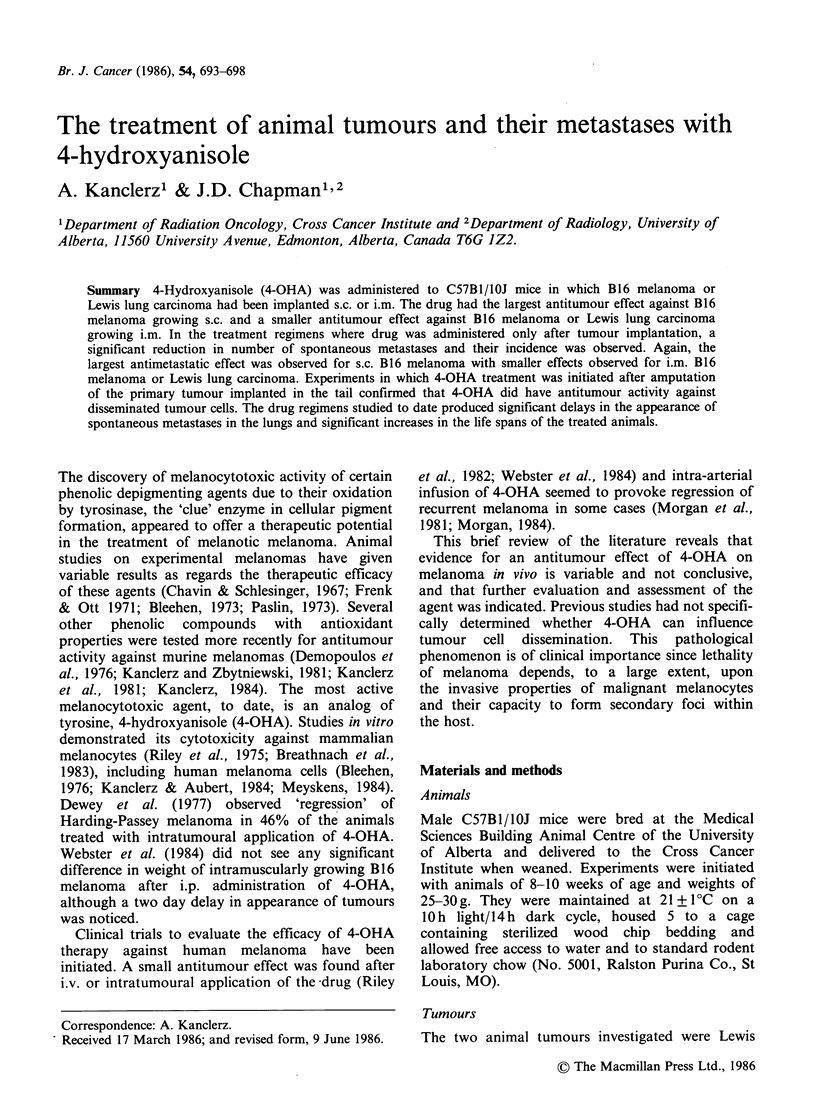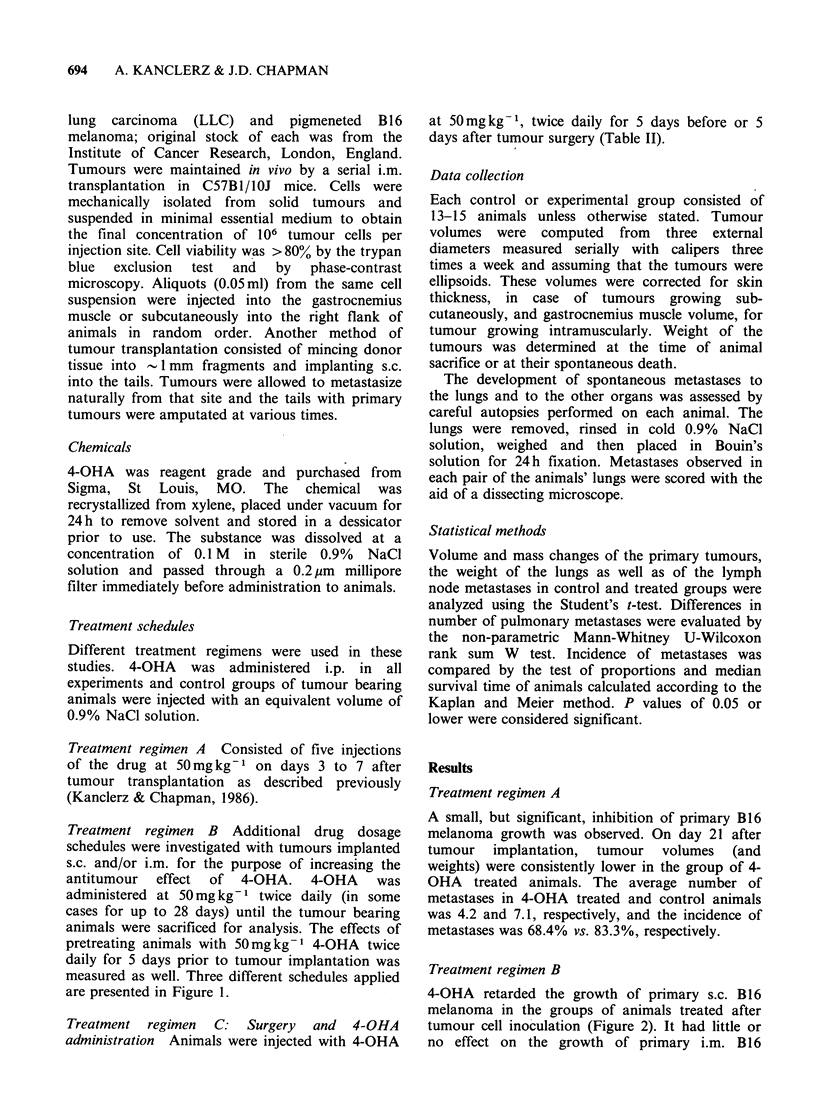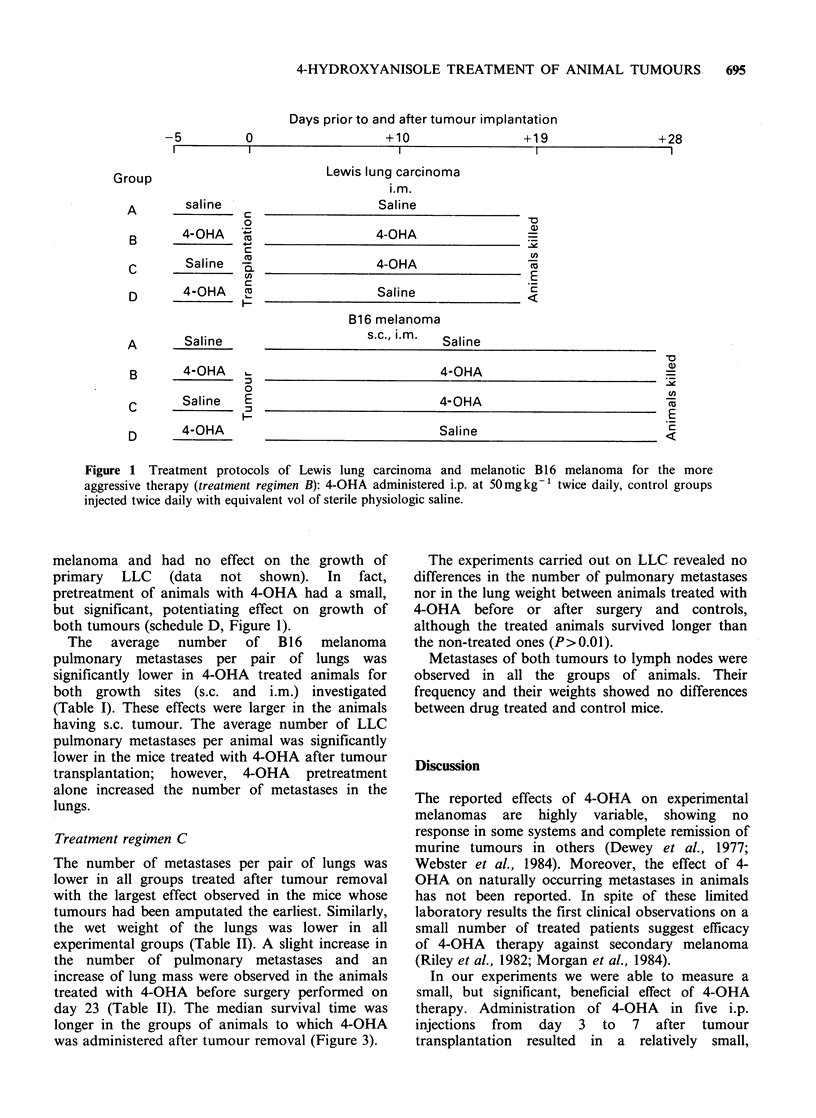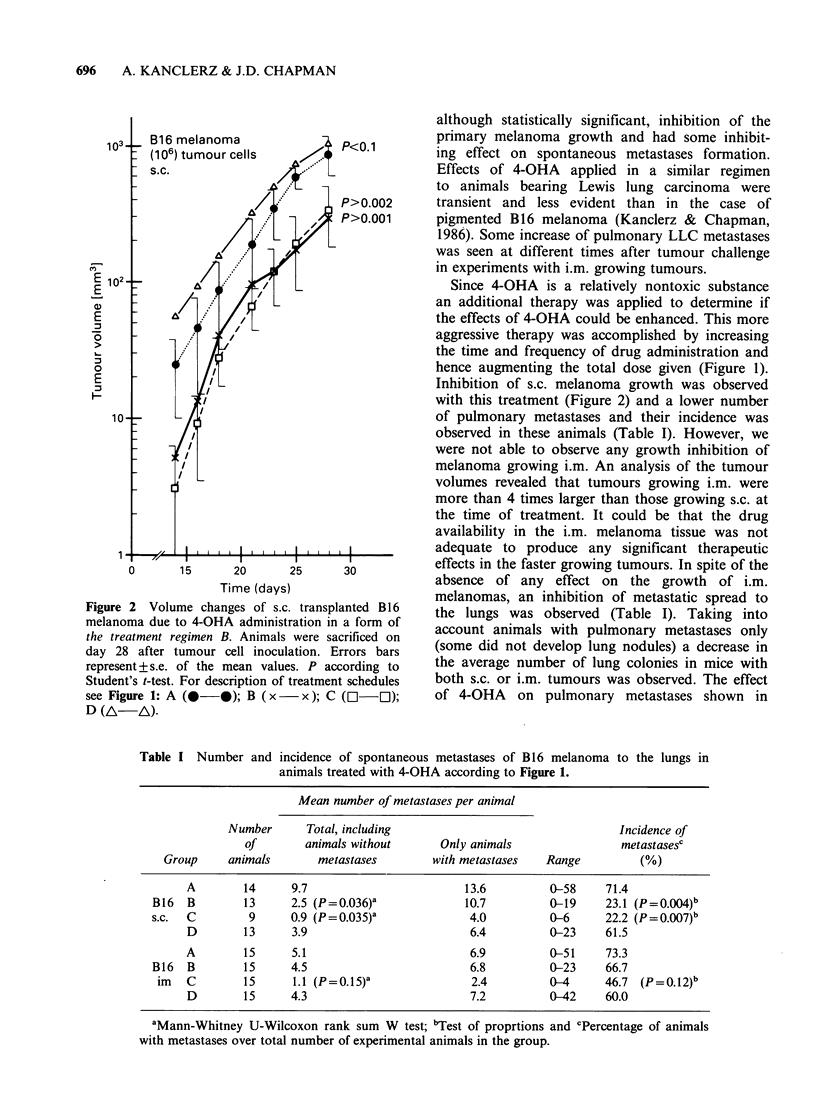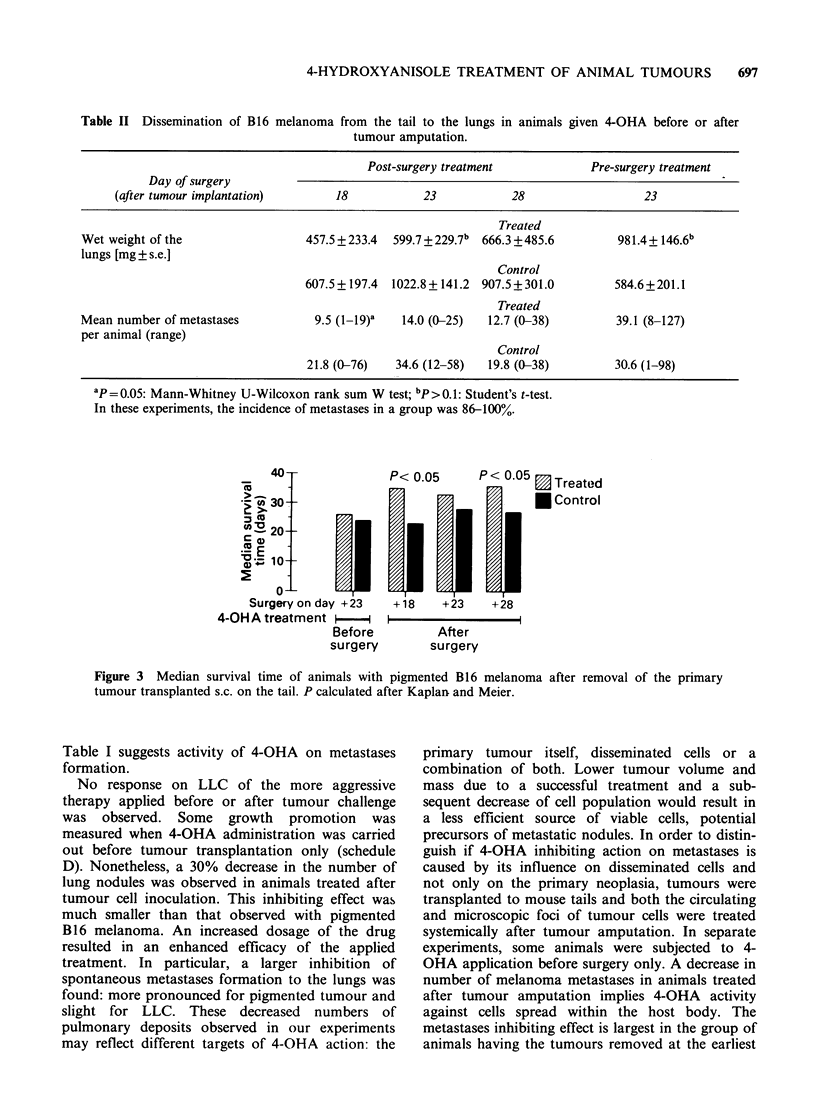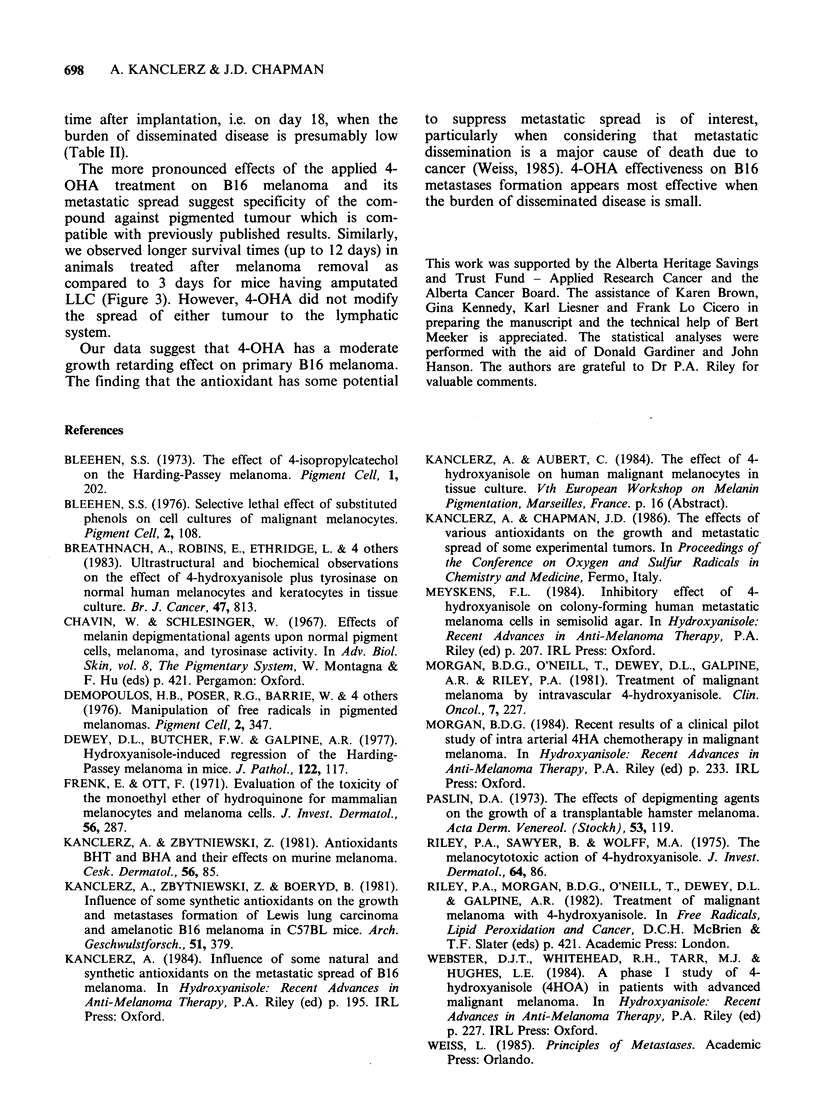# The treatment of animal tumours and their metastases with 4-hydroxyanisole.

**DOI:** 10.1038/bjc.1986.228

**Published:** 1986-10

**Authors:** A. Kanclerz, J. D. Chapman

## Abstract

4-Hydroxyanisole (4-OHA) was administered to C57Bl/10J mice in which B16 melanoma or Lewis lung carcinoma had been implanted s.c. or i.m. The drug had the largest antitumour effect against B16 melanoma growing s.c. and a smaller antitumour effect against B16 melanoma or Lewis lung carcinoma growing i.m. In the treatment regimens where drug was administered only after tumour implantation, a significant reduction in number of spontaneous metastases and their incidence was observed. Again, the largest antimetastatic effect was observed for s.c. B16 melanoma with smaller effects observed for i.m. B16 melanoma or Lewis lung carcinoma. Experiments in which 4-OHA treatment was initiated after amputation of the primary tumour implanted in the tail confirmed that 4-OHA did have antitumour activity against disseminated tumour cells. The drug regimens studied to date produced significant delays in the appearance of spontaneous metastases in the lungs and significant increases in the life spans of the treated animals.


*Br. J. Cancer* (1986), **54**, 693–698

### The treatment of animal tumours and their metastases with 4-hydroxyanisole

A. Kanclerz[1] & J.D. Chapman[1,2]

[1]*Department of Radiation Oncology, Cross Cancer Institute and* [2]*Department of Radiology, University of Alberta, 11560 University Avenue, Edmonton, Alberta, Canada T6G 1Z2.*

**Summary** 4-Hydroxyanisole (4-OHA) was administered to C57B1/10J mice in which B16 melanoma or Lewis lung carcinoma had been implanted s.c. or i.m. The drug had the largest antitumour effect against B16 melanoma growing s.c. and a smaller antitumour effect against B16 melanoma or Lewis lung carcinoma growing i.m. In the treatment regimens where drug was administered only after tumour implantation, a significant reduction in number of spontaneous metastases and their incidence was observed. Again, the largest antimetastatic effect was observed for s.c. B16 melanoma with smaller effects observed for i.m. B16 melanoma or Lewis lung carcinoma. Experiments in which 4-OHA treatment was initiated after amputation of the primary tumour implanted in the tail confirmed that 4-OHA did have antitumour activity against disseminated tumour cells. The drug regimens studied to date produced significant delays in the appearance of spontaneous metastases in the lungs and significant increases in the life spans of the treated animals.

The discovery of melanocytotoxic activity of certain phenolic depigmenting agents due to their oxidation by tyrosinase, the 'clue' enzyme in cellular pigment formation, appeared to offer a therapeutic potential in the treatment of melanotic melanoma. Animal studies on experimental melanomas have given variable results as regards the therapeutic efficacy of these agents (Chavin & Schlesinger, 1967; Frenk & Ott 1971; Bleehen, 1973; Paslin, 1973). Several other phenolic compounds with antioxidant properties were tested more recently for antitumour activity against murine melanomas (Demopoulos *et al.*, 1976; Kanclerz and Zbytniewski, 1981; Kanclerz *et al.*, 1981; Kanclerz, 1984). The most active melanocytotoxic agent, to date, is an analog of tyrosine, 4-hydroxyanisole (4-OHA). Studies *in vitro* demonstrated its cytotoxicity against mammalian melanocytes (Riley *et al.*, 1975; Breathnach *et al.*, 1983), including human melanoma cells (Bleehen, 1976; Kanclerz & Aubert, 1984; Meyskens, 1984). Dewey *et al.* (1977) observed 'regression' of Harding-Passey melanoma in 46% of the animals treated with intratumoural application of 4-OHA. Webster *et al.* (1984) did not see any significant difference in weight of intramuscularly growing B16 melanoma after i.p. administration of 4-OHA, although a two day delay in appearance of tumours was noticed.

Clinical trials to evaluate the efficacy of 4-OHA therapy against human melanoma have been initiated. A small antitumour effect was found after i.v. or intratumoural application of the drug (Riley *et al.*, 1982; Webster *et al.*, 1984) and intra-arterial infusion of 4-OHA seemed to provoke regression of recurrent melanoma in some cases (Morgan *et al.*, 1981; Morgan, 1984).

This brief review of the literature reveals that evidence for an antitumour effect of 4-OHA on melanoma *in vivo* is variable and not conclusive, and that further evaluation and assessment of the agent was indicated. Previous studies had not specifically determined whether 4-OHA can influence tumour cell dissemination. This pathological phenomenon is of clinical importance since lethality of melanoma depends, to a large extent, upon the invasive properties of malignant melanocytes and their capacity to form secondary foci within the host.

### Materials and methods

### *Animals*

Male C57B1/10J mice were bred at the Medical Sciences Building Animal Centre of the University of Alberta and delivered to the Cross Cancer Institute when weaned. Experiments were initiated with animals of 8–10 weeks of age and weights of 25–30 g. They were maintained at $21 \pm 1°C$ on a 10 h light/14 h dark cycle, housed 5 to a cage containing sterilized wood chip bedding and allowed free access to water and to standard rodent laboratory chow (No. 5001, Ralston Purina Co., St Louis, MO).

### *Tumours*

The two animal tumours investigated were Lewis

Correspondence: A. Kanclerz.
Received 17 March 1986; and revised form, 9 June 1986.

© The Macmillan Press Ltd., 1986

lung carcinoma (LLC) and pigmeneted B16 melanoma; original stock of each was from the Institute of Cancer Research, London, England. Tumours were maintained *in vivo* by a serial i.m. transplantation in C57Bl/10J mice. Cells were mechanically isolated from solid tumours and suspended in minimal essential medium to obtain the final concentration of $10^6$ tumour cells per injection site. Cell viability was >80% by the trypan blue exclusion test and by phase-contrast microscopy. Aliquots (0.05 ml) from the same cell suspension were injected into the gastrocnemius muscle or subcutaneously into the right flank of animals in random order. Another method of tumour transplantation consisted of mincing donor tissue into ~1 mm fragments and implanting s.c. into the tails. Tumours were allowed to metastasize naturally from that site and the tails with primary tumours were amputated at various times.

### Chemicals

4-OHA was reagent grade and purchased from Sigma, St Louis, MO. The chemical was recrystallized from xylene, placed under vacuum for 24 h to remove solvent and stored in a dessicator prior to use. The substance was dissolved at a concentration of 0.1 M in sterile 0.9% NaCl solution and passed through a 0.2 $\mu$m millipore filter immediately before administration to animals.

### Treatment schedules

Different treatment regimens were used in these studies. 4-OHA was administered i.p. in all experiments and control groups of tumour bearing animals were injected with an equivalent volume of 0.9% NaCl solution.

*Treatment regimen A* Consisted of five injections of the drug at 50 mg kg$^{-1}$ on days 3 to 7 after tumour transplantation as described previously (Kanclerz & Chapman, 1986).

*Treatment regimen B* Additional drug dosage schedules were investigated with tumours implanted s.c. and/or i.m. for the purpose of increasing the antitumour effect of 4-OHA. 4-OHA was administered at 50 mg kg$^{-1}$ twice daily (in some cases for up to 28 days) until the tumour bearing animals were sacrificed for analysis. The effects of pretreating animals with 50 mg kg$^{-1}$ 4-OHA twice daily for 5 days prior to tumour implantation was measured as well. Three different schedules applied are presented in Figure 1.

*Treatment regimen C: Surgery and 4-OHA administration* Animals were injected with 4-OHA at 50 mg kg$^{-1}$, twice daily for 5 days before or 5 days after tumour surgery (Table II).

### Data collection

Each control or experimental group consisted of 13–15 animals unless otherwise stated. Tumour volumes were computed from three external diameters measured serially with calipers three times a week and assuming that the tumours were ellipsoids. These volumes were corrected for skin thickness, in case of tumours growing subcutaneously, and gastrocnemius muscle volume, for tumour growing intramuscularly. Weight of the tumours was determined at the time of animal sacrifice or at their spontaneous death.

The development of spontaneous metastases to the lungs and to the other organs was assessed by careful autopsies performed on each animal. The lungs were removed, rinsed in cold 0.9% NaCl solution, weighed and then placed in Bouin's solution for 24 h fixation. Metastases observed in each pair of the animals' lungs were scored with the aid of a dissecting microscope.

### Statistical methods

Volume and mass changes of the primary tumours, the weight of the lungs as well as of the lymph node metastases in control and treated groups were analyzed using the Student's *t*-test. Differences in number of pulmonary metastases were evaluated by the non-parametric Mann-Whitney U-Wilcoxon rank sum W test. Incidence of metastases was compared by the test of proportions and median survival time of animals calculated according to the Kaplan and Meier method. *P* values of 0.05 or lower were considered significant.

### Results

### Treatment regimen A

A small, but significant, inhibition of primary B16 melanoma growth was observed. On day 21 after tumour implantation, tumour volumes (and weights) were consistently lower in the group of 4-OHA treated animals. The average number of metastases in 4-OHA treated and control animals was 4.2 and 7.1, respectively, and the incidence of metastases was 68.4% *vs.* 83.3%, respectively.

### Treatment regimen B

4-OHA retarded the growth of primary s.c. B16 melanoma in the groups of animals treated after tumour cell inoculation (Figure 2). It had little or no effect on the growth of primary i.m. B16

**Figure 1** Treatment protocols of Lewis lung carcinoma and melanotic B16 melanoma for the more aggressive therapy (*treatment regimen B*): 4-OHA administered i.p. at 50 mg $kg^{-1}$ twice daily, control groups injected twice daily with equivalent vol of sterile physiologic saline.

melanoma and had no effect on the growth of primary LLC (data not shown). In fact, pretreatment of animals with 4-OHA had a small, but significant, potentiating effect on growth of both tumours (schedule D, Figure 1).

The average number of B16 melanoma pulmonary metastases per pair of lungs was significantly lower in 4-OHA treated animals for both growth sites (s.c. and i.m.) investigated (Table I). These effects were larger in the animals having s.c. tumour. The average number of LLC pulmonary metastases per animal was significantly lower in the mice treated with 4-OHA after tumour transplantation; however, 4-OHA pretreatment alone increased the number of metastases in the lungs.

### *Treatment regimen C*

The number of metastases per pair of lungs was lower in all groups treated after tumour removal with the largest effect observed in the mice whose tumours had been amputated the earliest. Similarly, the wet weight of the lungs was lower in all experimental groups (Table II). A slight increase in the number of pulmonary metastases and an increase of lung mass were observed in the animals treated with 4-OHA before surgery performed on day 23 (Table II). The median survival time was longer in the groups of animals to which 4-OHA was administered after tumour removal (Figure 3).

The experiments carried out on LLC revealed no differences in the number of pulmonary metastases nor in the lung weight between animals treated with 4-OHA before or after surgery and controls, although the treated animals survived longer than the non-treated ones ($P > 0.01$).

Metastases of both tumours to lymph nodes were observed in all the groups of animals. Their frequency and their weights showed no differences between drug treated and control mice.

### Discussion

The reported effects of 4-OHA on experimental melanomas are highly variable, showing no response in some systems and complete remission of murine tumours in others (Dewey *et al.*, 1977; Webster *et al.*, 1984). Moreover, the effect of 4-OHA on naturally occurring metastases in animals has not been reported. In spite of these limited laboratory results the first clinical observations on a small number of treated patients suggest efficacy of 4-OHA therapy against secondary melanoma (Riley *et al.*, 1982; Morgan *et al.*, 1984).

In our experiments we were able to measure a small, but significant, beneficial effect of 4-OHA therapy. Administration of 4-OHA in five i.p. injections from day 3 to 7 after tumour transplantation resulted in a relatively small,

**Figure 2** Volume changes of s.c. transplanted B16 melanoma due to 4-OHA administration in a form of *the treatment regimen B.* Animals were sacrificed on day 28 after tumour cell inoculation. Errors bars represent ± s.e. of the mean values. *P* according to Student's *t*-test. For description of treatment schedules see **Figure 1**: A (●—●); B (×—×); C (□—□); D (△—△).

although statistically significant, inhibition of the primary melanoma growth and had some inhibiting effect on spontaneous metastases formation. Effects of 4-OHA applied in a similar regimen to animals bearing Lewis lung carcinoma were transient and less evident than in the case of pigmented B16 melanoma (Kanclerz & Chapman, 1986). Some increase of pulmonary LLC metastases was seen at different times after tumour challenge in experiments with i.m. growing tumours.

Since 4-OHA is a relatively nontoxic substance an additional therapy was applied to determine if the effects of 4-OHA could be enhanced. This more aggressive therapy was accomplished by increasing the time and frequency of drug administration and hence augmenting the total dose given (Figure 1). Inhibition of s.c. melanoma growth was observed with this treatment (Figure 2) and a lower number of pulmonary metastases and their incidence was observed in these animals (Table I). However, we were not able to observe any growth inhibition of melanoma growing i.m. An analysis of the tumour volumes revealed that tumours growing i.m. were more than 4 times larger than those growing s.c. at the time of treatment. It could be that the drug availability in the i.m. melanoma tissue was not adequate to produce any significant therapeutic effects in the faster growing tumours. In spite of the absence of any effect on the growth of i.m. melanomas, an inhibition of metastatic spread to the lungs was observed (Table I). Taking into account animals with pulmonary metastases only (some did not develop lung nodules) a decrease in the average number of lung colonies in mice with both s.c. or i.m. tumours was observed. The effect of 4-OHA on pulmonary metastases shown in

**Table I** Number and incidence of spontaneous metastases of B16 melanoma to the lungs in animals treated with 4-OHA according to **Figure 1.**

| | | | *Mean number of metastases per animal* | | | |
|---|---|---|---|---|---|---|
| *Group* | | *Number of animals* | *Total, including animals without metastases* | *Only animals with metastases* | *Range* | *Incidence of metastases*[c] (%) |
| | A | 14 | 9.7 | 13.6 | 0–58 | 71.4 |
| B16 | B | 13 | 2.5 ($P=0.036$)[a] | 10.7 | 0–19 | 23.1 ($P=0.004$)[b] |
| s.c. | C | 9 | 0.9 ($P=0.035$)[a] | 4.0 | 0–6 | 22.2 ($P=0.007$)[b] |
| | D | 13 | 3.9 | 6.4 | 0–23 | 61.5 |
| | A | 15 | 5.1 | 6.9 | 0–51 | 73.3 |
| B16 | B | 15 | 4.5 | 6.8 | 0–23 | 66.7 |
| im | C | 15 | 1.1 ($P=0.15$)[a] | 2.4 | 0–4 | 46.7 ($P=0.12$)[b] |
| | D | 15 | 4.3 | 7.2 | 0–42 | 60.0 |

[a]Mann-Whitney U-Wilcoxon rank sum W test; [b]Test of proprtions and [c]Percentage of animals with metastases over total number of experimental animals in the group.

**Table II** Dissemination of B16 melanoma from the tail to the lungs in animals given 4-OHA before or after tumour amputation.

| *Day of surgery (after tumour implantation)* | *Post-surgery treatment* 18 | 23 | 28 | *Pre-surgery treatment* 23 |
|---|---|---|---|---|
| | | | *Treated* | |
| Wet weight of the lungs [mg ± s.e.] | 457.5 ± 233.4 | 599.7 ± 229.7[b] | 666.3 ± 485.6 | 981.4 ± 146.6[b] |
| | | | *Control* | |
| | 607.5 ± 197.4 | 1022.8 ± 141.2 | 907.5 ± 301.0 | 584.6 ± 201.1 |
| | | | *Treated* | |
| Mean number of metastases per animal (range) | 9.5 (1–19)[a] | 14.0 (0–25) | 12.7 (0–38) | 39.1 (8–127) |
| | | | *Control* | |
| | 21.8 (0–76) | 34.6 (12–58) | 19.8 (0–38) | 30.6 (1–98) |

[a]$P = 0.05$: Mann-Whitney U-Wilcoxon rank sum W test; [b]$P > 0.1$: Student's *t*-test.
In these experiments, the incidence of metastases in a group was 86–100%.

**Figure 3** Median survival time of animals with pigmented B16 melanoma after removal of the primary tumour transplanted s.c. on the tail. *P* calculated after Kaplan and Meier.

Table I suggests activity of 4-OHA on metastases formation.

No response on LLC of the more aggressive therapy applied before or after tumour challenge was observed. Some growth promotion was measured when 4-OHA administration was carried out before tumour transplantation only (schedule D). Nonetheless, a 30% decrease in the number of lung nodules was observed in animals treated after tumour cell inoculation. This inhibiting effect was much smaller than that observed with pigmented B16 melanoma. An increased dosage of the drug resulted in an enhanced efficacy of the applied treatment. In particular, a larger inhibition of spontaneous metastases formation to the lungs was found: more pronounced for pigmented tumour and slight for LLC. These decreased numbers of pulmonary deposits observed in our experiments may reflect different targets of 4-OHA action: the primary tumour itself, disseminated cells or a combination of both. Lower tumour volume and mass due to a successful treatment and a subsequent decrease of cell population would result in a less efficient source of viable cells, potential precursors of metastatic nodules. In order to distinguish if 4-OHA inhibiting action on metastases is caused by its influence on disseminated cells and not only on the primary neoplasia, tumours were transplanted to mouse tails and both the circulating and microscopic foci of tumour cells were treated systemically after tumour amputation. In separate experiments, some animals were subjected to 4-OHA application before surgery only. A decrease in number of melanoma metastases in animals treated after tumour amputation implies 4-OHA activity against cells spread within the host body. The metastases inhibiting effect is largest in the group of animals having the tumours removed at the earliest time after implantation, i.e. on day 18, when the burden of disseminated disease is presumably low (Table II).

The more pronounced effects of the applied 4-OHA treatment on B16 melanoma and its metastatic spread suggest specificity of the compound against pigmented tumour which is compatible with previously published results. Similarly, we observed longer survival times (up to 12 days) in animals treated after melanoma removal as compared to 3 days for mice having amputated LLC (Figure 3). However, 4-OHA did not modify the spread of either tumour to the lymphatic system.

Our data suggest that 4-OHA has a moderate growth retarding effect on primary B16 melanoma. The finding that the antioxidant has some potential to suppress metastatic spread is of interest, particularly when considering that metastatic dissemination is a major cause of death due to cancer (Weiss, 1985). 4-OHA effectiveness on B16 metastases formation appears most effective when the burden of disseminated disease is small.

This work was supported by the Alberta Heritage Savings and Trust Fund – Applied Research Cancer and the Alberta Cancer Board. The assistance of Karen Brown, Gina Kennedy, Karl Liesner and Frank Lo Cicero in preparing the manuscript and the technical help of Bert Meeker is appreciated. The statistical analyses were performed with the aid of Donald Gardiner and John Hanson. The authors are grateful to Dr P.A. Riley for valuable comments.

### References

BLEEHEN, S.S. (1973). The effect of 4-isopropylcatechol on the Harding-Passey melanoma. *Pigment Cell*, **1**, 202.

BLEEHEN, S.S. (1976). Selective lethal effect of substituted phenols on cell cultures of malignant melanocytes. *Pigment Cell*, **2**, 108.

BREATHNACH, A., ROBINS, E., ETHRIDGE, L. & 4 others (1983). Ultrastructural and biochemical observations on the effect of 4-hydroxyanisole plus tyrosinase on normal human melanocytes and keratocytes in tissue culture. *Br. J. Cancer*, **47**, 813.

CHAVIN, W. & SCHLESINGER, W. (1967). Effects of melanin depigmentational agents upon normal pigment cells, melanoma, and tyrosinase activity. In *Adv. Biol. Skin, vol. 8, The Pigmentary System*, W. Montagna & F. Hu (eds) p. 421. Pergamon: Oxford.

DEMOPOULOS, H.B., POSER, R.G., BARRIE, W. & 4 others (1976). Manipulation of free radicals in pigmented melanomas. *Pigment Cell*, **2**, 347.

DEWEY, D.L., BUTCHER, F.W. & GALPINE, A.R. (1977). Hydroxyanisole-induced regression of the Harding-Passey melanoma in mice. *J. Pathol.*, **122**, 117.

FRENK, E. & OTT, F. (1971). Evaluation of the toxicity of the monoethyl ether of hydroquinone for mammalian melanocytes and melanoma cells. *J. Invest. Dermatol.*, **56**, 287.

KANCLERZ, A. & ZBYTNIEWSKI, Z. (1981). Antioxidants BHT and BHA and their effects on murine melanoma. *Cesk. Dermatol.*, **56**, 85.

KANCLERZ, A., ZBYTNIEWSKI, Z. & BOERYD, B. (1981). Influence of some synthetic antioxidants on the growth and metastases formation of Lewis lung carcinoma and amelanotic B16 melanoma in C57BL mice. *Arch. Geschwulstforsch.*, **51**, 379.

KANCLERZ, A. (1984). Influence of some natural and synthetic antioxidants on the metastatic spread of B16 melanoma. In *Hydroxyanisole: Recent Advances in Anti-Melanoma Therapy*, P.A. Riley (ed) p. 195. IRL Press: Oxford.

KANCLERZ, A. & AUBERT, C. (1984). The effect of 4-hydroxyanisole on human malignant melanocytes in tissue culture. *Vth European Workshop on Melanin Pigmentation, Marseilles, France*. p. 16 (Abstract).

KANCLERZ, A. & CHAPMAN, J.D. (1986). The effects of various antioxidants on the growth and metastatic spread of some experimental tumors. In *Proceedings of the Conference on Oxygen and Sulfur Radicals in Chemistry and Medicine*, Fermo, Italy.

MEYSKENS, F.L. (1984). Inhibitory effect of 4-hydroxyanisole on colony-forming human metastatic melanoma cells in semisolid agar. In *Hydroxyanisole: Recent Advances in Anti-Melanoma Therapy*, P.A. Riley (ed) p. 207. IRL Press: Oxford.

MORGAN, B.D.G., O'NEILL, T., DEWEY, D.L., GALPINE, A.R. & RILEY, P.A. (1981). Treatment of malignant melanoma by intravascular 4-hydroxyanisole. *Clin. Oncol.*, **7**, 227.

MORGAN, B.D.G. (1984). Recent results of a clinical pilot study of intra arterial 4HA chemotherapy in malignant melanoma. In *Hydroxyanisole: Recent Advances in Anti-Melanoma Therapy*, P.A. Riley (ed) p. 233. IRL Press: Oxford.

PASLIN, D.A. (1973). The effects of depigmenting agents on the growth of a transplantable hamster melanoma. *Acta Derm. Venereol. (Stockh)*, **53**, 119.

RILEY, P.A., SAWYER, B. & WOLFF, M.A. (1975). The melanocytotoxic action of 4-hydroxyanisole. *J. Invest. Dermatol.*, **64**, 86.

RILEY, P.A., MORGAN, B.D.G., O'NEILL, T., DEWEY, D.L. & GALPINE, A.R. (1982). Treatment of malignant melanoma with 4-hydroxyanisole. In *Free Radicals, Lipid Peroxidation and Cancer*, D.C.H. McBrien & T.F. Slater (eds) p. 421. Academic Press: London.

WEBSTER, D.J.T., WHITEHEAD, R.H., TARR, M.J. & HUGHES, L.E. (1984). A phase I study of 4-hydroxyanisole (4HOA) in patients with advanced malignant melanoma. In *Hydroxyanisole: Recent Advances in Anti-Melanoma Therapy*, P.A. Riley (ed) p. 227. IRL Press: Oxford.

WEISS, L. (1985). *Principles of Metastases*. Academic Press: Orlando.